# Motorized Recreation Sounds Influence Nature Scene Evaluations: The Role of Attitude Moderators

**DOI:** 10.3389/fpsyg.2018.00495

**Published:** 2018-04-13

**Authors:** Jacob Benfield, B. D. Taff, David Weinzimmer, Peter Newman

**Affiliations:** ^1^Penn State Abington, Abington Township, PA, United States; ^2^Recreation, Park, and Tourism Management, Pennsylvania State University, University Park, PA, United States; ^3^Human Dimensions of Natural Resources, Colorado State University, Fort Collins, CO, United States

**Keywords:** noise, affect, resource management, national park, overflight, motorcycle

## Abstract

Soundscape assessment takes many forms, including letting the consequences of the soundscape be an indicator of soundscape quality or value. As a result, much social science research has been conducted to better quantify problem soundscapes and the subsequent effects on humans exposed to them. Visual evaluations of natural environments are one area where research has consistently shown detrimental effects of noisy or anthropogenic soundscapes (e.g., those containing noise from motorized recreation), but the potential moderating role of individual attitudes toward elements within the soundscape has not been sufficiently explored. This study demonstrates that both pro-motorized recreation and pro-motorized recreation management attitudes can alter the effect of motorized recreation noise on scenic evaluations in opposing directions. Pro-recreation attitudes lessen the effect of the soundscape, while pro-management attitudes heighten the negative effect of anthropogenic sounds on scenic evaluation. The implications for other areas of soundscape research, especially with regard to soundscape quality assessment through experienced outcomes, are discussed, including possible strategies for prioritizing known or relevant moderating variables.

## Introduction

Soundscapes represent a dynamic, complex system of auditory stimuli that can encompass both objective and subjective properties (Bell et al., 2001). For instance, an outdoor concert held at the Acropolis in Athens, Greece creates a soundscape rich with physical stimuli. These include elements such as pitch or intensity, both able to be measured via instrumentation of varied types, while the content of the music itself, combined with the historic location, can embody more subjective properties, such as joy or sorrow, to those in attendance. The measurement of that subjective experience and the assessment of how the soundscape drives the effect utilize completely different methods and instrumentation. As such, the scientific study of soundscapes can be equally as dynamic and complex as the study of visual landscapes.

For example, some research on soundscape focuses heavily on urban soundscapes or transportation effects on residential soundscapes with varying degrees of objective physical measurement or more subjective qualitative interviewing (e.g., Botteldooren et al., 2006; Payne, 2008; Jeon et al., 2010; Craig et al., 2014). Others have emphasized natural or rural soundscape assessment with varying levels of instrumentation, acoustical monitoring, or survey sampling (e.g., De Coensel and Botteldooren, 2006; Stack et al., 2011; Reed et al., 2012; Jiang et al., 2017). Each of these studies, with varied approaches and built or largely natural environments, contributes to our understanding of soundscapes in meaningful ways but all utilized different assessment methods.

The current study represents one approach—utilizing laboratory simulations of experimentally manipulated soundscapes in a visual landscape evaluation task—aimed at the assessment of subjective qualities and outcomes of soundscapes within a specific type of environment (i.e., protected natural areas). In other words, one method for assessing soundscapes relies less on the physical qualities of the soundscape itself but instead on the outcomes that occur during or immediately following exposure to that soundscape. In this study, the outcome of interest is changes in perception of the visual landscape that occur under differing soundscapes. Perhaps most importantly, this study aims also to highlight an aspect of subjective survey-based soundscape assessment that often goes unstudied or unreported: the moderating role of individual attitudes toward the stimuli.

### Natural Soundscapes as a Resource and Management Priority

Existing soundscape research in natural areas supports the idea that opportunities to experience the sounds of nature are important factors in determining the quality of recreational experiences to visitors of these areas. Foundational research by Driver et al. (1987) demonstrated that in choosing their experiences many outdoor recreationists are motivated to find respite from excessive noise and urban environments. Subsequently, McDonald et al. (1995) found that the vast majority (over 90%) of survey respondents in national parks and protected areas listed the enjoyment of natural quiet and the sounds of nature as important reasons for their visit. In recent years, it has become increasingly apparent to land managers that natural soundscapes are as deserving of protection and careful stewardship as other natural and cultural resources (Newman et al., 2010).

In the United States, federal land management policies over the last few decades have responded to the concern that natural areas are threatened by growing levels of noise from human activities and development. In 1987, the U.S. National Parks Overflights Act was a pioneering piece of legislation that sought for the first time to systematically protect natural soundscapes in National Park Service (NPS) lands. According to Gramann (1999), this law provided the impetus for new directions of investigation into the effects of noise in parks and other protected areas.

The NPS addressed soundscape management directly in 2000 by Director’s Order #47 (“Soundscape Preservation and Noise Management”), with the goal “to articulate National Park Service operational policies that will require, to the fullest extent practicable, the protection, maintenance, or restoration of the natural soundscape resource in a condition unimpaired by inappropriate or excessive noise sources” (National Park Service [NPS], 2000, p. 1). This order sought to establish official direction for the preservation of intrinsic park soundscapes by means of better planning, monitoring, and assessment. Also in 2000, the National Parks Air Tour Management Act mandated that the NPS and Federal Aviation Administration work together to identify and mitigate adverse effects of commercial air tours on the soundscapes of parks (National Park Service [NPS], 2000). In 2006, the NPS re-affirmed its commitment to restoring and protecting natural soundscapes by addressing soundscape management in several sections of its official Management Policies, including the statement that “The Service will preserve, to the greatest extent possible, the natural soundscapes of parks” (National Park Service [NPS], 2006, section 4.9). Similar mandates designed to better protect or manage natural soundscapes or to mitigate excessive noise have occurred in many other countries (e.g., Directive 2002/49/EC of the European Parliament, 2017). Thus, the need for clear management standards and mechanisms for assessing soundscape quality and impact in a range of contexts becomes not only a research issue, but a societal one as well.

### Laboratory Research on Soundscapes Assessment for Natural Environments

An important consequence of these legislative and administrative mandates is the pursuit of biological, physical, and social science research on the existing soundscape as well as effects of soundscape on inhabitants and users of natural areas. In other words, these mandates have helped to generate a need for soundscape assessment across several domains of study. One area that has been of particular interest to social scientists and recreation researchers has been the role of natural and anthropogenic soundscapes on visitor evaluations of natural scenes and areas. For example, motorized recreation in the form of snowmobiles, propeller plane overflights, all-terrain vehicle (ATV) excursions, and organized motorcycle rallies are common activities in natural areas but all generate large amounts of high intensity, disruptive anthropogenic noise into the soundscape. Visitors to those locations who are not participating in those activities may have a lessened experience because of that additional vehicle noise. While field-based assessments have also been conducted (e.g., Pilcher et al., 2009; Marin et al., 2011), laboratory simulations have been equally useful and prevalent in assessing the perceived quality and impact of different soundscapes.

For example, early research by Mace et al. (1999) adapted the traditional laboratory-based landscape assessment paradigm to test for impacts from motorized recreation noise on aesthetic ratings of landscape quality in simulated national park settings. This laboratory study looked at potential effects of helicopter tour noise on evaluations of scenic overviews in Grand Canyon National Park. In addition to collecting aesthetic ratings, self-reported changes in affective state in response to the auditory stimuli were also collected. Results of this study showed that negative experiential and aesthetic effects were associated with soundscapes that included helicopter noise, relative to purely natural soundscapes. This was one of the first studies to directly demonstrate that auditory environments could influence visual ratings.

Follow-up research by Mace et al. (2003) investigated the importance of soundscape source attribution when assessing the presence of motorized helicopter noise in natural settings. To test this, the researchers presented the same auditory stressor (i.e., helicopter overflights) within the soundscape but attributed its purpose to either scenic overflights for tourists, backcountry maintenance activities by park management, or life-saving search and rescue operations. The results indicated that a soundscape dominated by helicopter noise from either tourist overflights or official park management activities was similarly detrimental to the experiences of potential park visitors when compared to natural-only soundscapes. It was also shown that type of visual scenery, such as mountainous or forested, also partially determined the amount of influence different soundscapes had on ratings.

Subsequent laboratory work by Benfield et al. (2010) expanded the findings of Mace et al. (1999, 2003) in several ways. First, the range of soundscape elements investigated was expanded from helicopter noise to include human voices, airplane overflights, and motorized ground vehicles. Second, the range of soundscape settings evaluated was expanded to include three additional national parks (Yellowstone, Everglades, and Olympic National Parks) to demonstrate the robustness of the previously shown location effect. Finally, the number of affective outcomes assessed was expanded to include fatigue, hostility, and other specific states beyond positive or negative affect. Consistent with the prior studies, the anthropogenic soundscapes were each responsible for detriments to both affective state and visual assessment of the landscapes shown. Individual positive affect, attentiveness, and serenity was lowered by the presence of anthropogenic soundscapes, and ratings of hostility increased. Visual assessments of scenic tranquility, beauty and solitude similarly decreased in the presence of anthropogenic noise while ratings of annoyance were higher compared to those in the natural sound condition.

Most recently, Weinzimmer et al. (2014) further refined the assessment of specific soundscape events by comparing directly three common sources of motorized noise in national parks – motorcycles, propeller planes, and snowmobiles. Using a carefully controlled laboratory simulation, Weinzimmer et al. (2014) directly compared different motorized vehicle soundscapes using a within-subjects design. Those direct comparisons replicated previous studies by showing that motorized recreation noise had significant, detrimental effects on both aesthetic and affective dimensions. Those comparisons also demonstrated interesting differences between the three different sources of anthropogenic noise showing that nuanced assessments of the qualities of the sounds themselves were needed.

### Potential Moderators of Subjective Soundscape Assessments

In addition to assessing the main effect of anthropogenic sound on visual evaluations or self-reported mood, research in this domain has occasionally examined different moderators of the effect soundscape has on those outcomes. Some of those efforts have been more successful than others.

As stated previously, Mace et al. (2003) manipulated sound exposure but also examined the role of sound source attribution on subsequent scenic evaluations. By describing the helicopter noise as arising from either legitimate park operations (maintenance, search, and rescue) or from tourist entertainment (scenic overflight), the authors hoped to show that higher perceived legitimacy of the noise source would lessen the detrimental effect previously observed. While subtle differences were shown between the different noise attributions (e.g., scenic beauty was lower for legitimate conditions compared to tourist activity), the overwhelming consensus was that the presence of helicopter noise was detrimental irrespective of attribution given for the sound.

Benfield et al. (2010) conducted *ad hoc* analyses to test the interaction between the visual appeal of the scene being assessed and the soundscape of the scene. They showed that more beautiful scenes, as assessed by participants in the absence of sound, were more affected by the presence of anthropogenic sounds than scenes rated as less beautiful. This effect was shown to generalize across sound types (voice, aircraft, and ground traffic) and the four different parks tested. Essentially, this moderation effect when combined with the overall findings suggested that soundscapes could impact visual quality assessment but that the inherent visual quality of the scene also determined the magnitude of impact any given sound could have on the ratings.

Despite these advances in understanding potential moderators that may influence subjective perceptions of soundscape and subsequent impacts that soundscape has on other scene ratings, much less research has examined how individual attitudes – positive or negative – toward specific elements in the soundscape change those outcomes. This is in spite of a wealth of research on attitudes affecting other aspects of natural resource assessment or management.

Manfredo et al. (2004) suggested that attitudes are some of the most frequently examined and central measures within the assessment of human dimensions of natural resources. For example, over the past 30 years attitudes have been found to predict and influence support of recreational management strategies (Bright, 1997), preferences toward national forest use and management strategies (Clement and Cheng, 2011), perceptions of crowding (Shelby and Heberlein, 1986; Manning, 2007), evaluations of wildlife management strategies (Manfredo, 2008), use of transportation in parks (White et al., 2011; Taff et al., 2013), and perceptions toward resource impacts (Monz, 2009), to name a few. However, only a few studies have explored attitudes toward noise sources and soundscape assessment specifically.

Within the urban setting of Hong Kong, Lam et al. (2009) found that negative attitudes toward railway noise increased annoyance of associated soundscapes but did not significantly affect annoyance toward road-based traffic noise. In the Netherlands, Pedersen et al. (2009) found that negative attitudes toward the visual impact of wind turbines significantly increased annoyance from turbine-associated noise. Tarrant et al. (1995) conducted surveys with visitors to Wyoming wilderness areas to explore attitudes toward seeing and hearing aircraft, as well as other dimensions of wilderness experience. The authors found that respondents’ estimates of noise levels were strongly related to their attitudes toward aircraft overflights, suggesting that wilderness visitors may respond differently to aircraft based in part on their attitudes.

Lai et al. (2009) segmented visitors to a national seashore based on their attitudes toward natural resource management in order to develop marketing strategies. One of the attitudinal items they evaluated related to the elimination of human-caused noises from the seashore, which factored into a dimension the authors termed ‘preventing encroachment.’ This study discovered three different visitor segments based on respondent attitudes, which included ‘conservation-oriented,’ ‘development-oriented,’ and ‘status quo’ visitors. Results indicated that conservation-oriented respondents were most supportive of ‘preventing encroachment,’ while development-oriented respondents were least supportive of this action.

Finally, Taff et al. (2014) indirectly manipulated park visitor attitudes toward an existing noise source through the use of messaging. Specifically, prior research had shown that aircraft overflights in a national park in the western United States from a nearby military installation were both frequently noticed and consistently rated as detrimental to the visitor experience. As a follow-up to that finding, these researchers asked park visitors to rate the acceptability of several sound clips taken from inside the park, with some containing a higher prevalence and intensity of military aircraft noise. Half of the surveyed visitors were given no information about the clips while the other half were given information about the overflights’ purpose, including the overflights being “in an effort to help keep the United States of America safe.” Participants in the “keeping America safe condition” were less likely to rate the overflights as problematic or below minimal levels of acceptability than those who heard the soundscapes without context.

Attitudes are among the most important measures when determining management approaches in parks and protected areas (Manfredo et al., 2004; Vaske, 2008) and should thus be included in the assessment of soundscapes in those areas. However, the potential moderating role of attitudes toward recreation or recreation management within the context of park soundscape experiences deserves additional attention. Specifically, research has not assessed how attitudes toward motorized recreation or the management of motorized recreational noise influences the evaluation of park soundscape experiences.

### The Current Study

Research consistently shows that soundscapes dominated by anthropogenic stimuli has a detrimental effect on visual evaluations of natural landscapes (Mace et al., 1999). That same research has shown that situational aspects, such as noise source attribution (Mace et al., 2003) or the beauty of the scene being evaluated (Benfield et al., 2010), can moderate the effect that sound can have on subsequent evaluations. However, attitudes of the person experiencing the noise and making the evaluations have not been adequately explored as potential moderators. Considering other research has shown that individual attitudes can impact individual perception of both anthropogenic noise (Taff et al., 2014) and a host of other management policies (Manfredo et al., 2004), a better understanding of the effect of attitudes in relation to recreation noise and soundscape assessment is warranted.

To test the potential moderating role of recreation or management attitudes, an experimental laboratory simulation similar to those cited previously was carried out. Individual attitudes in favor of motorized recreation (i.e., “pro-recreation” attitudes) or in favor of the regulation of motorized recreational noise (i.e., “pro-management” attitudes) were assessed prior to the simulation and the following hypotheses were made:

H1: Individuals with a higher pro-recreation attitude will be less affected by a recreation noise soundscape when making scene evaluations or reporting affective state following exposure to recreation noise.
H2: Individuals with a higher pro-management attitude will be more affected by a recreation noise soundscape when making scene evaluations or reporting affective state following exposure to recreation noise.

## Materials and Methods

### Participants

Seventy-seven undergraduate and graduate students (43 females and 34 males) participated in a laboratory-based study for course research credit. Participants were of mostly of typical college age (*M* = 22.38 years; *SD* = 6.89; range = 16–50) and reported regular visits to national parks within the previous 12 months (*M* = 2.94, *SD* = 1.40).

### Design

This study utilized a 2 (soundscape) × 3 (park setting) repeated measures design. Participants received both soundscape conditions (natural only, natural and motorized recreation noise) in a randomized order. Within each of the soundscape conditions, participants viewed images of three different national park settings (Yellowstone, Glacier, and Denali) in random order. For analysis purposes, scenic evaluations are aggregated across parks and comparisons are made between the aggregate scene score for the two soundscape conditions.

### Materials and Measures

#### Scenic Evaluations

Scenic evaluations were based on an existing landscape assessment paradigm adapted for use in soundscape research (e.g., Mace et al., 1999; Benfield et al., 2010). Evaluations were performed along eight dimensions of aesthetic quality: naturalness, freedom, preference, annoyance, solitude, scenic beauty, tranquility, and acceptability. Prior researchers had chosen these dimensions to incorporate both physical qualities of the scene (e.g., beauty, naturalness) as well as affordances within in (e.g., solitude, freedom) and were retained in this study to allow for direct comparisons with the most relevant prior literature. Ratings were obtained on a 10-point scale ranging from “1 = very low” to “10 = very high” and a composite score for the eight dimensions was used for all analyses (the annoyance dimension was reverse-coded prior to analysis). Participants were instructed to “evaluate the scene you viewed on the following characteristics” which emphasizes visual perceptions but encompasses the entirety of the scene, including the auditory stimuli.

#### Recreation Management Attitudes

Recreation management attitudes were measured using a set of six items intended to measure project relevant attitudes along two opposing dimensions – acceptability of motorized recreation in spite of noise (e.g., “I would be willing to take a motorcycle through a park even if I knew the noise bothered other visitors.”; three items; α = 0.84) and acceptability of banning motorized recreational vehicles (e.g., “Snowmobiles should not be allowed in national parks due to the noise they create.”; three items; α = 0.86). These items were created based on interview responses of different user groups (motorized recreation users and pro-motorized management users) of one of the parks tested. These two scales, while conceptually representing two related but opposing viewpoints, were judged to be separate from one another. The two scales were negatively correlated with one another but at only moderate levels (*r* = -0.57). Factor analysis of those six items revealed two separate factors, representing 60.81% (pro-motorized recreation) and 16.82% (pro-motorized management) of the total variance. Further, factor loadings for each scale were strong for included items (>0.8) and weak for items in the other scale (<0.29). Within each dimension, items were rated along a seven-point “1 = strongly disagree” to “7 = strongly agree” continuum with an average response across items being calculated.

#### Affective Ratings

Affective ratings were collected by self-report using the 20-item Positive and Negative Affect Scale (PANAS; Watson et al., 1988). Prior research (Mace et al., 1999; Benfield et al., 2010) had utilized the PANAS or its extended version and had shown that motorized sounds can also impact affective state of those making the ratings. Again, these measures were retained in this study to allow for direct comparisons with the most relevant prior literature that had not explored the role of moderators. Participants completed the PANAS at three different time points during the experimental procedure: at baseline, following the first natural sound condition, and following the first motorized sound condition. The PANAS consists of a series of words that represent different feelings, and participants use a five-point scale (ranging from 1 = “very slightly or not at all” to 5 = “extremely”) to report how much each word describes how they are feeling at that moment. Half of the items are combined to give a positive affect score (α = 0.86–0.91; such as “enthusiastic,” “determined,” and “interested”), while the other half are combined to provide a negative affect score (α = 0.79–0.89; examples include “upset,” “nervous,” and “irritable”).

#### Soundscape Conditions

Soundscape conditions consisted of three conditions with only natural sounds (i.e., birds, wind, and water), and three conditions with motorized sounds added to the natural soundtracks. The motorized sounds consisted of recordings of a propeller plane, snowmobile, and a pair of motorcycles. All sound clips were obtained from the actual parks they were designed to represent in the simulations. Each participant experienced all six conditions (three with natural sounds only; three with overlaid motorized sounds) in one of six pseudo-randomized orders. Sound clips were 45 s in duration, with 7-s fade-in and fade-out effects. All clips were normalized such that the three natural clips had equivalent sound energy levels and the three motorized clips had equivalent sound energy levels. The normalized sound clips were then calibrated so that participants would hear (via headphones) the natural clips at approximately 45 dB(A) and the motorized clips at approximately 60 dB(A); these sound levels were chosen to be representative of those regularly experienced in these locations by visitors.

#### Visual Scene Stimuli

Visual scene stimuli were chosen from available landscape photographs of three popular national parks: Yellowstone National Park, Glacier National Park, and Denali National Park and Preserve. These parks were chosen because of on-going and publicly debated issues of motorized recreation management in these areas. Photographic scenes were selected as representative scenic views within the parks. Developments like roads and buildings were not visible in the scenes. Four photographs from each park were included in the experiment, as well as two practice scenes from Grand Canyon National Park. Summer scenes were chosen for Glacier and Denali, while winter scenes were selected for Yellowstone. Seasons corresponded to the specific source of motorized noise from each park (e.g., winter scenes to match snowmobile noise in Yellowstone).

### Procedure

Participants were recruited from courses at a large state funded, public university located in close proximity (45 miles) to a large U.S. National Park. Responses were collected on iPad second generation computers (Apple Inc., Cupertino, CA, United States) programmed with iSURVEY software (Contact Software Limited, Wellington, New Zealand). Prior to participation in the research, all participants provided written consent to participate based on an IRB approved study description.

The landscape assessment task consisted of eight blocks of scenic ratings: two practice blocks, three natural sound blocks, and three motorized recreation sound blocks. Within each block, participants rated four visual scenes from the same park while being exposed to a single soundscape condition. These scenes were shown in random order for 45 s each and participants began making evaluations after 20 s. Thus, the total block time was 3 min of exposure to a single soundscape across four scenes; each block produced four sets of scenic evaluations along the eight aesthetic dimensions.

**FIGURE 1:**
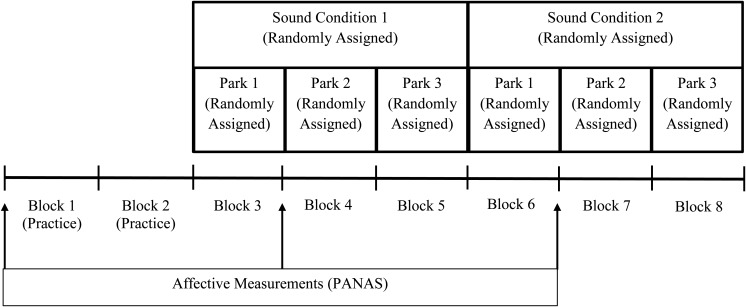
Procedural diagram for landscape evaluation task and affective measures.

Affective states from the PANAS were acquired before the two practice blocks, following the first natural sounds block, and following the first motorized sounds block. This spacing of the PANAS measurement (i.e., every three blocks) corresponds to approximately 9min between each measurement with the most recent 3 min including the corresponding soundscape of interest (baseline, natural, or motorized). The order of the natural or motorized sets of blocks was also randomized. See **Figure 1** for a summary of the procedure.

## Results

The purpose of this study was to examine the potential moderating role that individual attitudes could have on the previously observed relationship between soundscape type and scenic evaluations or affective state. To test this, the average score on attitude items was used as a covariate in repeated measures analysis of covariance (R-ANCOVAs) comparing scene ratings or affective state between natural and motorized soundscape exposures (see **Tables 1**, **2**).

**Table 1:** Results of repeated measure ANCOVA comparing natural and anthropogenic noise with pro-motorized recreation attitude covariate.

Outcome		*F*	$\eta_{p}^{2}$
**Pro-recreation attitude**			
Scenic evaluations	Sound type	201.83**	0.734
	Attitude	3.96*	0.051
	Sound type × attitude	10.04**	0.121
Change in positive affect	Affect change	7.65**	0.097
	Sound type	0.53	0.007
	Attitude	3.49^†^	0.047
	Affect × sound type	23.74**	0.251
	Affect × attitude	1.05	0.015
	Sound type × attitude	0.15	0.002
	Affect × sound type × attitude	7.60**	0.097
Change in negative affect	Affect change	4.76*	0.063
	Sound type	3.97*	0.053
	Attitude	4.11*	0.055
	Affect × sound type	11.37**	0.138
	Affect × attitude	6.49*	0.084
	Sound type × attitude	1.17	0.016
	Affect × sound type × attitude	2.38	0.032


*^†^*p* < 0.08, ^∗^*p* < 0.05, ^∗∗^*p* < 0.01.*

**Table 2:** Results of repeated measure ANCOVA comparing natural and anthropogenic noise with pro-management of motorized recreation attitude covariate.

Outcome		*F*	$\eta_{p}^{2}$
**Pro-management attitude**			
Scenic evaluations	Sound type	30.75**	0.296
	Attitude	3.85*	0.050
	Sound type × attitude	13.12**	0.152
Change in positive affect	Affect change	0.44	0.006
	Sound type	1.74	0.024
	Attitude	0.26	0.004
	Affect × sound type	0.01	0.000
	Affect × attitude	4.53*	0.060
	Sound type × attitude	2.60	0.035
	Affect × sound Type × attitude	4.22*	0.056
Change in negative affect	Affect change	3.51^†^	0.047
	Sound type	0.23	0.003
	Attitude	9.31	0.116
	Affect × sound type	0.01	0.000
	Affect × attitude	3.52^†^	0.047
	Sound type × attitude	1.79	0.025
	Affect × sound Type × attitude	2.61	0.035


*^†^*p* < 0.08, ^∗^*p* < 0.05, ^∗∗^*p* < 0.01.*

### Results for Scenic Evaluation

Consistent with prior research (e.g., Mace et al., 1999; Benfield et al., 2010), a main effect for sound condition on scenic evaluations was shown for both attitude moderators in the ANCOVAs. Specifically, scene ratings were higher for the natural sound condition (*M* = 9.19, *SD* = 0.60) when compared to scene ratings from motorized soundscapes (*M* = 5.94, *SD* = 1.31) for both the pro-motorized recreation attitude model [*F*(1,73) = 201.83, *p* < 0.01, $\eta_{p}^{2}$ = 0.734] and the pro-management attitude model [*F*(1,73) = 30.75, *p* < 0.01, $\eta_{p}^{2}$ = 0.296].

**FIGURE 2:**
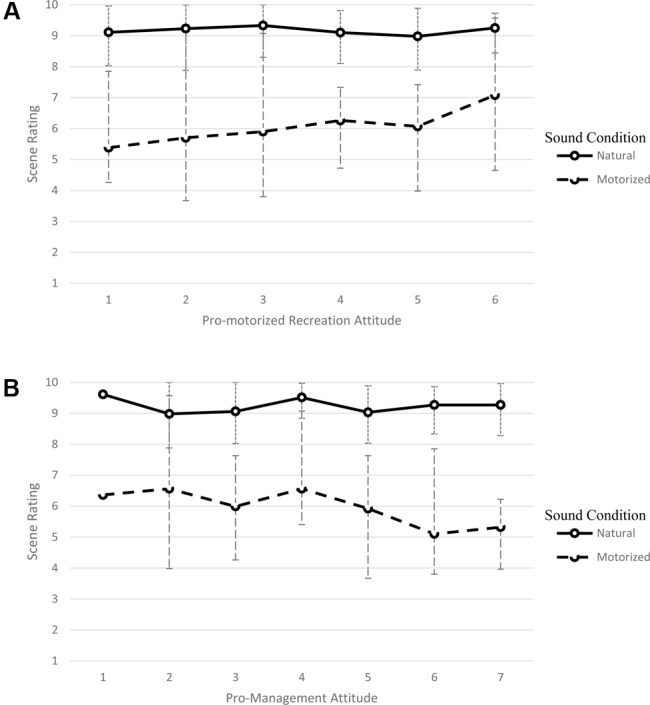
Moderating effect of **(A)** pro-motorized recreation attitudes or **(B)** pro-management of motorized recreation attitudes on composite scene evaluations in the presence of natural or anthropogenic sounds. Bars represent the minimum and maximum values for scene rating observed for participants with that corresponding attitude score.

Consistent with study hypotheses, a significant interaction between sound type and the attitude moderator was also shown for both the pro-recreation attitude score [*F*(1,73) = 10.04, *p* = 0.002, $\eta_{p}^{2}$ = 0.121] and the pro-management attitude score [*F*(1,73) = 13.12, *p* = 0.001, $\eta_{p}^{2}$ = 0.152]. As predicted, higher pro-recreation attitudes lessened the negative effect of motorized sound on composite scene ratings (**Figure 2A**). The opposite pattern was found for pro-management attitudes: higher pro-management attitudes increased the negative effect of motorized sound on composite ratings (**Figure 2B**). **Table 1** displays the summary results for both repeated measure ANCOVAs.

### Results for Affective Ratings

Positive and negative affect were assessed both before and after each of the sound exposures, and repeated measures ANCOVA was used to test the potential moderating role of attitude on affective state. For each analysis, pre- and post-exposure PANAS ratings were analyzed for each sound condition with the attitude variable added as a covariate. Thus, a significant three-way interaction between the change in affect rating, the sound condition, and the attitude measure indicated that attitude was moderating the effect on affective state for some sound conditions but not others. Main effects and two-way interactions were of less relevance to the current study aims and not hypothesized, although the interaction between change in affect and sound condition would be consistent with previous research (e.g., Mace et al., 1999; Benfield et al., 2010).

#### Pro-motorized Recreation Attitudes

Pro-motorized recreation attitudes were hypothesized to lessen the previously shown deleterious effect of recreation noise on both positive and negative affect (e.g., *less* decrease in positive affect and *less* increase in negative affect). **Table 1** displays the full model results for pro-motorized recreation attitudes as a moderating variable.

For positive affect, full model analyses of the pro-motorized recreation attitude data showed main effects for change in positive affect after exposure [*F*(1,71) = 7.65, *p* = 0.007, $\eta_{p}^{2}$ = 0.097], but no main effect for sound condition [*F*(1,71) = 0.53, *p* = 0.469]. However, a two-way interaction between affect and sound condition [*F*(1,71) = 23.74, *p* < 0.001, $\eta_{p}^{2}$ = 0.251] indicated that affect scores changed following exposure but differentially depending on sound condition and consistent with prior research. The interaction between change in positive affect and attitude score was also not significant [*F*(1,71) = 1.04, *p* = 0.310]. As hypothesized, the full three-way interaction between change in affect, sound condition, and pro-motorized recreation attitude score was significant indicating that attitude was moderating the previously observed effect that soundscape can have on positive affect, *F*(1,71) = 7.60, *p* = 0.007, $\eta_{p}^{2}$ = 0.097.

**FIGURE 3:**
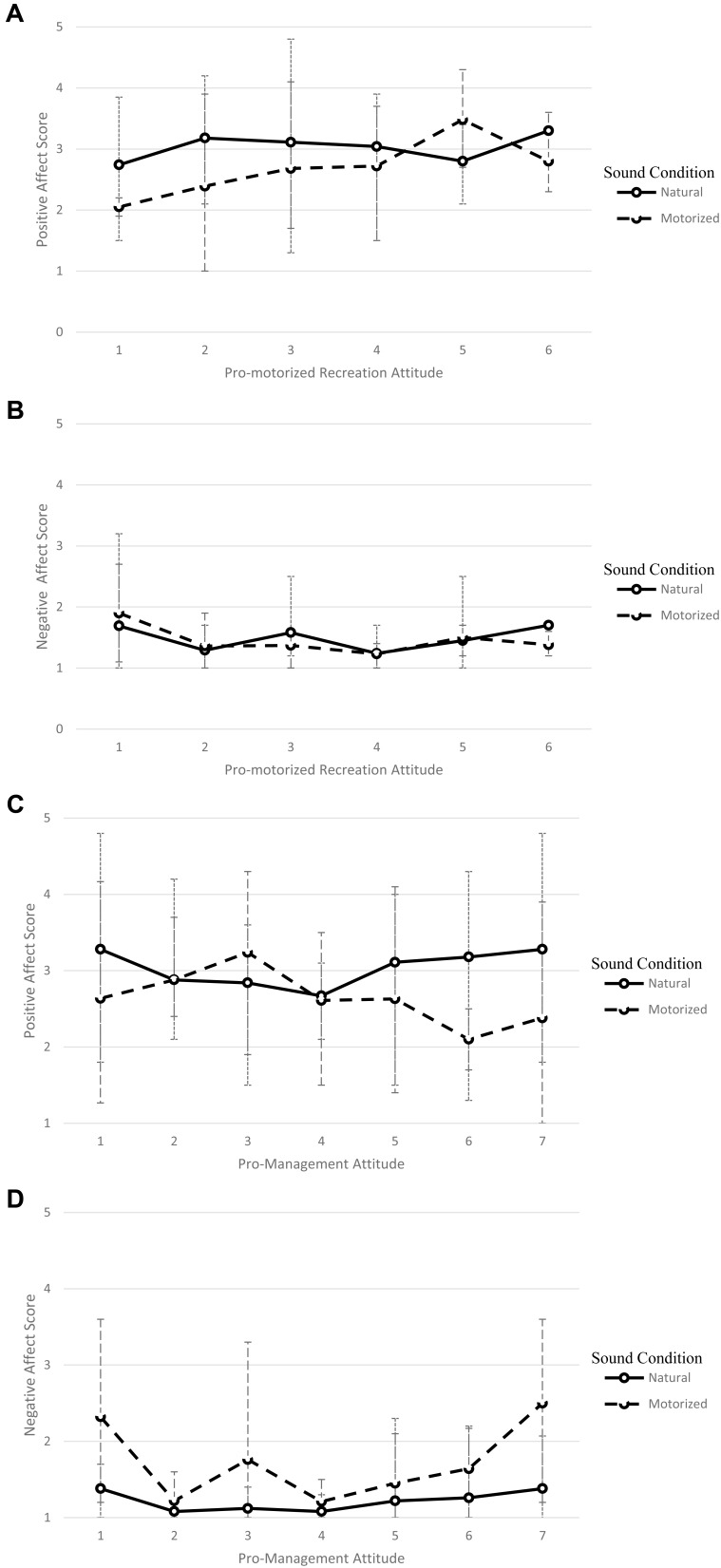
Moderating effect of pro-motorized recreation attitudes on positive affect **(A)** and negative affect **(B)** or pro-management of motorized recreation attitudes on positive affect **(C)** and negative affect **(D)** in the presence of natural or anthropogenic sounds. Bars represent the minimum and maximum values for scene rating observed for participants with that corresponding attitude score.

Follow-up analyses to unpack that three-way interaction were conducted (**Table 3**). Consistent with the hypothesis, pro-recreation attitudes were shown to significantly interact with positive affect ratings for the recreation noise condition (*F* = 7.73, *p* = 0.009, η^2^ = 0.181) but not the natural sound condition (*F* = 1.40, *p* = 0.244). Those with higher pro-motorized recreation attitudes showed less decrease in positive affect following exposure to motorized recreation noise in the scene (**Figure 3A**).

**Table 3:** Results of repeated measures ANCOVAs for natural and motorized sound exposure conditions separately showing interactions between affect ratings and attitude scores.

		*F*	$\eta_{p}^{2}$
**Pro-recreation attitude: positive affect**				
Natural	Affect	2.38	0.062
	Attitude	0.93	0.025
	Affect × attitude	1.40	0.037
Motorized	Affect	27.26**	0.438
	Attitude	3.07^†^	0.081
	Affect × attitude	7.73**	0.181
**Pro-recreation attitude: negative affect**			
Natural	Affect	0.99	0.027
	Attitude	0.51	0.014
	Affect × attitude	0.61	0.017
Motorized	Affect	11.73**	0.251
	Attitude	4.45*	0.113
	Affect × attitude	7.36**	0.174
**Pro-management attitude: positive affect**			
Natural	Affect	0.17	0.005
	Attitude	2.24	0.059
	Affect × attitude	0.00	0.000
Motorized	Affect	0.28	0.008
	Attitude	0.61	0.017
	Affect × attitude	8.55**	0.196
**Pro-management attitude: negative affect**			
Natural	Affect	2.15	0.056
	Attitude	1.82	0.048
	Affect × attitude	0.05	0.001
Motorized	Affect	1.53	0.042
	Attitude	7.97**	0.185
	Affect × attitude	4.63*	0.117


*^†^*p* < 0.08, ^∗^*p* < 0.05, ^∗∗^*p* < 0.01.*

Results for negative affect were similarly consistent with previous research and hypotheses (**Figure 3B**). There was a main effect for change in negative affect following exposure [*F*(1,71) = 4.76, *p* = 0.032] and for sound condition [*F*(1,71) = 3.97, *p* = 0.050]. Additional two-way interactions between change in affect and sound condition [*F*(1,71) = 11.37, *p* = 0.001] and change in affect and attitude [*F*(1,71) = 6.49, *p* = 0.013] were also shown to be significant. In those interactions, negative affect increased after exposure to the motorized sound but not natural sound.

The hypothesized three-way interaction between negative affect, sound condition, and attitude did not reach significance [*F*(1,71) = 2.38, *p* = 0.128]. However, separate R-ANCOVAs, similar to those performed to better understand the significant three-way interaction for positive affect, did show the same pattern of significant two-way interactions between pro-recreation attitude and negative affect ratings following recreation noise (*F* = 7.36, *p* = 0.010, η^2^ = 0.174) but not natural sounds (*F* = 0.61, *p* = 0.438). Thus, the primary analyses for negative affect failed to show the same hypothesized effect on the three-way interaction, but simpler analyses of the two-way interactions did mirror those shown for positive affect (**Table 3**).

#### Pro-management

Pro-management attitudes were hypothesized to interact with both PANAS positive and negative affect scores but in a manner contrary to pro-recreation attitudes; it was hypothesized that greater pro-management attitudes would relate to larger affective change in the presence of motorized recreation noise but not in the presence of natural sounds (**Table 2**).

For positive affect, full model analyses of the pro-management attitude data showed no main effects for change in positive affect after exposure [*F*(1,71) = 0.44, *p* = 0.508], sound condition [*F*(1,71) = 1.74, *p* = 0.192], or the attitude moderator [*F*(1,71) = 0.26, *p* = 0.612]. However, a two-way interaction between affect and the attitude moderator [*F*(1,71) = 4.53, *p* < 0.037, $\eta_{p}^{2}$ = 0.060] indicated that affect scores changed following exposure but differentially depending on the attitude moderator. The interaction between change in positive affect and sound condition was also not significant [*F*(1,71) = 0.01, *p* = 0.943]. However, as hypothesized, the full three-way interaction between change in affect, sound condition, and pro-management of motorized recreation attitude score was significant indicating that attitude was moderating the observed effect that soundscape can have on positive affect, *F*(1,71) = 4.22, *p* = 0.044, $\eta_{p}^{2}$ = 0.056.

Follow-up results were consistent with the hypothesis for recreational noise exposure and pro-management attitudes (**Table 3**). When exposed to motorized recreation noise, a significant interaction between change in positive affect and the attitude moderator was present, *F*(1,71) = 8.55, *p* < 0.001, $\eta_{p}^{2}$ = 0.196. The observed decrease in positive affect following exposure to motorized recreation noise was larger for those with high pro-management attitudes (**Figure 3C**). The same interaction and moderating relationship was not present when participants were exposed to natural sounds, *F*(1,71) = 0.00, *p* = 0.958. In short, the moderating effect of pro-management attitudes on change in positive affect following exposure to motorized recreation noise was observed, as hypothesized.

For negative affect, the full model results were not supportive of hypotheses (**Figure 3D**). The main effect for the attitude moderator was the only main effect or interaction shown in the analysis, *F*(1,71) = 9.31, *p* = 0.003, $\eta_{p}^{2}$ = 0.116. Similar to the negative affect results for the pro-motorized recreation analyses, simple analyses with two R-ANCOVAs, one for each sound condition, suggested differential effects of the attitude moderator on change in negative affect in the hypothesized manner (i.e., attitude moderation for recreation noise exposure but not for natural sound exposure).

In summary, the hypothesized moderating relationship between changes in affect following sound exposure and the type of sound presented was present for both the pro-recreation and the pro-management attitude moderators, but only for positive affect. In the case of negative affect, the hypothesized interaction failed to reach statistical significance in the full models but demonstrated some support when analyzing each sound condition in isolation (**Table 3**).

## Discussion

Previous research has consistently demonstrated a deleterious effect of anthropogenic noise on scenic evaluations and affect (e.g., Mace et al., 1999; Weinzimmer et al., 2014), but the examination of moderating variables within this type of soundscape assessment has been limited to situational characteristics such as the cause (Mace et al., 2003) or location (Benfield et al., 2010) of the noise. The current study demonstrated that the individual-level characteristics of attitudes toward motorized recreation noise and soundscape management can also affect the severity of anthropogenic noise-related outcomes in simulated natural recreation environments.

Specifically, the data presented show that pro-motorized recreation attitudes reduced the negative impact of motorized recreation noise on scenic evaluations and ratings of positive affective state. While the presence of the noise still reduced ratings of landscape quality and positive affect, the effect was much smaller for those high in pro-recreation attitudes compared to those with lower levels of the same attitude. The reverse was true of pro-soundscape management attitudes. The presence of motorized recreation noise was more problematic to those holding high pro-management attitudes than for those with a lesser extent of these attitudes. Pro-management attitudes predicted lower scene evaluations in the presence of recreation noise and larger changes in positive affect.

The moderating role of pro-management and pro-motorized recreation attitudes on subsequent perception and evaluation of outdoor recreation and leisure environments under varied sound conditions has not been demonstrated in prior research, thus representing an important addition to our current understanding of how the objectively measurable soundscape and the subjectively experiencing user interact in natural environments. The connection between this set of findings and other research (e.g., Tarrant et al., 1995; Clement and Cheng, 2011) ties the emerging area of natural soundscapes to a larger literature on attitudes in recreation enjoyment and management which has a number of implications for outcomes-based soundscape assessment for both researchers and managers alike.

### Implications

Recreation area managers need to make informed policy and management decisions that impact a wide range of user groups and the current project can aid those management efforts. In this management context, the assessment of soundscape quality often relies of user outcomes relative to competing economic or public goals. For example, the U.S. National Park Service must balance visitor use, which in some locations includes natural soundscape altering, noise-producing motorized recreational activities, while at the same time preserving the natural and cultural resources within these protected areas for visitors to enjoy separately from motorized recreation. Thus, in order to provide quality soundscape experiences while protecting park resources and outside economic interests, it is imperative that managers understand not only the overall soundscape experience but also who visitors are, their motivations, expectations, and how they perceive other aspects of the park experience. Much of this can be determined by assessing visitor attitudes toward recreational settings and management actions (Manfredo et al., 2004), and this study has provided greater understanding of these factors.

By understanding attitudes and the role they play in outcomes-based soundscape assessment, managers can use informational messaging to strengthen attitudes that align with management objectives (e.g., protection of natural sounds) or alter attitudes that misalign with management goals. The extensive body of persuasion literature suggests that effective messaging design requires consideration of many variables (e.g., personal relevance, message source, and timing) that are thought to enhance and motivate understanding in order to alter attitudinal state (Petty and Cacioppo, 1986; Fishbein and Manfredo, 1992; Eagly and Chaiken, 1993; Perloff, 2003; Absher and Bright, 2004). Messages that have the most effect on attitudes contain substantial argument quality, which is thought to stimulate elaboration (Petty and Cacioppo, 1986; Petty and Wegener, 1998; Wood, 2000). In other words, researchers and managers, by relying on a substantial body of literature on persuasion and attitude change, would be potentially able to alter the subjective assessment of a soundscape rather than altering the physical properties of the soundscape itself.

Interpretive strategists cannot reach or alter the attitudes of all visitors due to situational and/or personal variables, but developing messages that are strong, impactful, and relevant increases attitudinal change or strength (Ham, 2007). Attitudes that align with a message containing impactful arguments are thought to be strengthened, while misaligning attitudes may be altered, if a message enhances consideration and thought about a given topic (Lavine and Snyder, 1996; Wood, 2000; Ziegler et al., 2007; Petty and Wegener, 2008). With consideration of messaging strategies, these results suggest that specific messages emphasizing the impact of motorized recreation noise (e.g., disturbing wildlife or other visitors) could influence those individuals with pro-recreation attitudes to be more cognizant regarding the protection of natural sounds. Alternatively, those individuals with pro-management attitudes could experience strengthened attitudes by receiving this type of targeted message. Future laboratory and field studies related to messaging, perspective taking, or attitude change in the context of soundscape assessment would be justified given the current set of findings.

### Limitations

Participants in this study were not explicitly informed about the sources of noise that were presented within the soundscape, and the sources were not visible during the simulations. It is possible that some participants were not aware that they were hearing motorcycles, snowmobiles, and propeller planes, specifically. Furthermore, the noise sources were not clearly attributed to recreation activities, although other work suggests that attribution may not make a significant impact on assessment (e.g., Mace et al., 2003). Participants were not told that they would be hearing sounds from scenic air tours as opposed to commercial or park administrative flights (e.g., general maintenance or search and rescue operations), which could be perceived similarly but evaluated differently. While these factors can be considered limitations, it is quite possible that explicit attribution of the noise sources to recreational motorized activities would increase the magnitude of the observed effects reported above. For example, changes in landscape assessments and affective response could be underestimated for participants who hold strong attitudes about park management or motorized recreation, but who were not aware that they were hearing sounds generated by those activities.

However, other work has shown that laboratory-based soundscape assessments, particularly in the context of identification and representation, can lead to greater variability in reporting when compared to field-based soundscape assessments (e.g., Guastavino et al., 2005). Similarly, the evaluations taking place are derived from stimuli that is both visual and aural. As such, the landscape context, being natural and remote without the presence of built structures, informs expectations for those soundscapes and subsequent scenic assessments in their presence. Previous research has shown that such visual elements can impact noise and sound assessment, particularly on nature-relevant constructs such as those assessed in this study (e.g., Pheasant et al., 2008; Pheasant and Watts, 2015).

Another potential limitation of the study design relates to the lack of a direct link between recreation attitudes and participation in recreation activities. Participants were not asked to report if they had actually engaged in the motorized activities simulated in this study (or even if they intended to participate in the activities). Rather, they evaluated hypothetical scenarios of motorized vehicle use in national park settings. As discussed further in the next section, it would be informative to test actual members of motorized user groups, who are likely to have strong attitudes about motorized recreation in national parks to see if that indeed alters soundscape assessment. Similarly, the current sample consisted primarily of university students in natural resources classes. Based on their training, these students would be expected to exhibit a bias in favor of park management and resource protection. Natural resources students may also be more knowledgeable than typical park visitors about soundscape-related controversies, assessment strategies, and management objectives in protected areas. Finally, the age of participants may inform both their attitudes and overall response to motorized recreation noise. Younger people, such as the majority this sample, may have their hearing less impacted by external noise sources, view motorized recreation as more appealing, or have less experience in these types of environmental contexts.

Thus, it is reasonable to assume that a more representative sample of potential park visitors would demonstrate a wider range of attitudes and increase the external validity of the findings; however, the combination of laboratory and field-based methodologies offers the strongest approach for investigating the multi-dimensional impacts of motorized noise on visitors (Mace et al., 2013). The present study attempted to isolate through well-controlled experimental manipulation the individual psychological factors that moderate this outcomes-based assessment. The robustness of the observed effect should now be tested in other settings and with other samples of visitors, recreation managers, and non-visiting adults.

### Future Directions

In addition to research designed to address methodological limitations discussed above, the current study provides several avenues for additional research on soundscape assessment, generally. As mentioned previously, the connection between attitude and subsequent soundscape appreciation allows for the wealth of literature on attitude change and persuasion to be utilized as a mechanism for combating problematic noise and/or increasing enjoyment of unique or more pristine soundscapes. Such interventions would run counter to more physical properties-based soundscape assessments because they allow for altering outcomes without changing the actual stimuli.

Showing that individual attitudes can moderate the effect of soundscape on environmental assessment suggests that other individual features need to be more fully incorporated into soundscape assessment research and more fully considered when making management policy. While some work has been done with personality traits (e.g., Benfield et al., 2013), the same cannot be said of other individual visitor variables such as motivation. Research has already demonstrated that visitor motivations for quiet can alter the perceived acceptability of anthropogenic sounds (Marin et al., 2011), so it seems highly probable that such a motivation for quiet would also affect ratings of scenes in the presence of sounds or changes in affective state caused by the presence of those sounds. Research focused on motivations should be conducted to confirm that connection between acceptability and subsequent changes in scene ratings or affect.

Additionally, little research has effectively demonstrated that anthropogenic noise, in the specific context of natural environments, alters physiological processes related to arousal or stress. Such effects have been demonstrated in wildlife (e.g., Barber et al., 2010), but the connection to park visitors experiencing sounds has not been shown. It may be possible that such physiological effects within outcomes-based soundscape assessment would be moderated by attitude given that some attitudes, such as being in favor of motorized recreation, related to more positive affective responses in the current study. Similarly, emerging research has demonstrated a restorative effect of natural soundscapes (Alvarsson et al., 2010; Benfield et al., 2014; Abbott et al., 2016), but has not examined whether individual attitude, or another variable such as motivation, could moderate that restorative effect.

In summary, the current study showed that outcomes-based soundscape assessment would benefit from additional reliance and focus on moderating variables. In this case, pro-motorized recreation and or management attitudes moderate a well-established set of findings within soundscape assessment research. Such an effect had not been previously shown and, more importantly, has several implications for both management policy and future research as it pertains to soundscape assessment. Based on the current findings, it is reasonable to predict that attitudes may moderate other soundscape-relevant effects, and that other characteristics, such as motivations, may be worth examining in the future and controlling for when making assessment of soundscape quality based on user perceptions, reported experiences, or outcomes.

## Ethics Statement

This study was carried out in accordance with the recommendations of the American Psychological Association with written informed consent from all subjects in accordance with the Declaration of Helsinki and the Belmont Committee Report. The protocol was approved by the Internal Review Board at the university conducting the research.

## Author Contributions

JB conceptualized the RQ, conducted the analysis, and was the primary author of all content in the manuscript. BT contributed significantly to the literature review and analysis and was also the second highest contributor of manuscript content. DW conceptualized the original research project and collected the data. PN supervised DW’s data collection and assisted with editing of later drafts of the manuscript.

## Conflict of Interest Statement

The authors declare that the research was conducted in the absence of any commercial or financial relationships that could be construed as a potential conflict of interest.

## References

[B1] Abbott L. C. Taff B. D. Newman P. Benfield J. A. Mowen A. J. (2016). The influence of natural sounds on attention restoration. *J. Parks Recreation Adm.* 34 5–15. 10.18666/JPRA-2016-V34-I3-6893

[B2] Absher J. D. Bright A. D. (2004). “Communication research in outdoor recreation and natural resources management,” in *Society and Natural Resources: A Summary of Knowledge*, eds Manfredo M. J. Vaske J. J. Bruyere B. L. Field D. R. Brown P. J. (Jefferson City, MO: Modern Litho), 117–126.

[B3] Alvarsson J. J. Wiens S. Nilsson M. E. (2010). Stress recovery during exposure to nature sound and environmental noise. *Int. J. Environ. Res. Public Health* 7 1036–1046. 10.3390/ijerph7031036 20617017 PMC2872309

[B4] Barber J. R. Crooks K. R. Fristrup K. M. (2010). The costs of chronic noise exposure for terrestrial organisms. *Trends Ecol. Evol.* 25 180–189. 10.1016/j.tree.2009.08.002 19762112

[B5] Bell P. A. Greene T. C. Fisher J. D. Baum A. (2001). *Environmental Psychology*, 5th Edn London: Lawrence Erlbaum.

[B6] Benfield J. A. Bell P. A. Troup L. J. Soderstrom N. C. (2010). Aesthetic and affective effects of vocal and traffic nose on natural landscape assessment. *J. Environ. Psychol.* 30 103–111. 10.1016/j.jenvp.2009.10.002

[B7] Benfield J. A. Nurse G. A. Jakubowski R. Gibson A. W. Taff B. D. Newman P. (2013). Testing noise in the field: a brief measure of individual noise sensitivity. *Environ. Behav.* 46 353–372. 10.1177/0013916512454430

[B8] Benfield J. A. Taff B. D. Newman P. Smyth J. (2014). Natural sound facilitates mood recovery. *Ecopsychology* 6 183–188.

[B9] Botteldooren D. De Coensel B. De Muer T. (2006). The temporal structure of urban soundscapes. *J. Sound Vib.* 292 105–123. 10.1016/j.jsv.2005.07.026

[B10] Bright A. D. (1997). Attitude strength and support of recreation management strategies. *J. Leis. Res.* 29 363–379. 10.1080/00222216.1997.11949804

[B11] Clement J. M. Cheng A. S. (2011). Using analyses of public value orientations, attitudes and preferences to inform national forest planning in Colorado and Wyoming. *Appl. Geogr.* 31 393–400. 10.1016/j.apgeog.2010.10.001

[B12] Craig A. Knox D. Moore D. (2014). The perceived annoyance of urban soundscapes. *J. Acoust. Soc. Am.* 136 2305–2305. 10.1121/1.4900339

[B13] De Coensel B. Botteldooren D. (2006). The quiet rural soundscape and how to characterize it. *Acta Acust. United Acust.* 92 887–897.

[B14] Directive 2002/49/EC of the European Parliament (2017). *Directive 2002/49/EC of the European Parliament and of the Council of 25 June 2002 Relating to the Assessment and Management of Environmental Noise.* Available at: http://eur-lex.europa.eu/LexUriServ/LexUriServ.do?uri=CELEX:32002L0049:en:NOT [accessed November 22, 2017].

[B15] Driver B. L. Nash R. Haas G. (1987). “Wilderness benefits: a state-of-knowledge view,” in *Proceedings of the National Wilderness Research Conference: Issues, State of Knowledge, Future Directions*, Ogden, UT, 294–319.

[B16] Eagly A. H. Chaiken S. (1993). *The Psychology of Attitudes.* Fort Worth, TX: Harcourt Brace Jovanovich.

[B17] Fishbein M. Manfredo M. J. (1992). “A theory of behavior change,” in *Influencing Human Behavior: Theory and Application in Recreation, Tourism, and Natural Resources Management*, ed. Manfredo M. J. (Champaign, IL: Sagamore Publishing Inc.).

[B18] Gramann J. (1999). The effect of mechanical noise and natural sound on visitor experiences in units of the National Park System. *Soc. Sci. Res. Rev.* 1 1–16.

[B19] Guastavino C. Katz B. F. Polack J. D. Levitin D. J. Dubois D. (2005). Ecological validity of soundscape reproduction. *Acta Acust. United Acust.* 91 333–341.

[B20] Ham S. H. (2007). From interpretation to protection: Is there a theoretical basis? *J. Assoc. Herit. Interpret.* 12 20–23.

[B21] Jeon J. Y. Lee P. J. You J. Kang J. (2010). Perceptual assessment of quality of urban soundscapes with combined noise sources and water sounds. *J. Acoust. Soc. Am.* 127 1357–1366. 10.1121/1.3298437 20329835

[B22] Jiang J. Zhang J. Zhang H. Yan B. (2017). Natural soundscapes and tourist loyalty to nature-based tourism destinations: the mediating effect of tourist satisfaction. *J. Travel Tourism Mark.* 35 162–177.

[B23] Lai P.-H. Sorice M. G. Nepal S. K. Cheng C.-K. (2009). Integrating social marketing into sustainable resource management at Padre Island National Seashore: an attitude-based segmentation approach. *Environ. Manage.* 43 985–998. 10.1007/s00267-009-9293-9 19381715

[B24] Lam K.-C. Chan P.-K. Chan T.-C. Au W.-H. Hui W.-C. (2009). Annoyance response to mixed transportation noise in Hong Kong. *Appl. Acoust.* 70 1–10. 10.1016/j.apacoust.2008.02.005

[B25] Lavine H. Snyder M. (1996). Cognitive processing and the functional matching effect in persuasion: the mediating role of subjective perceptions of message quality. *J. Exp. Soc. Psychol.* 32 580–604. 10.1006/jesp.1996.0026 8979935

[B26] Mace B. L. Bell P. A. Loomis R. J. (1999). Aesthetic, affective, and cognitive effects of noise on natural landscape assessment. *Soc. Nat. Resour.* 12 225–242. 10.1080/089419299279713

[B27] Mace B. L. Bell P. A. Loomis R. J. Haas G. E. (2003). Source attribution of helicopter noise in pristine national park landscapes. *J. Park Recreation Adm.* 21 97–119.

[B28] Mace B. L. Corser G. C. Zitting L. Denison J. (2013). Effects of overflights on the national park experience. *J. Environ. Psychol.* 35 30–39. 10.1016/j.jenvp.2013.04.001

[B29] Manfredo M. J. (2008). *Who Cares about Wildlife: Social Science Concepts for Exploring Human-wildlife Relationships and Conservation Issues.* New York, NY: Springer Press 10.1007/978-0-387-77040-6

[B30] Manfredo M. J. Teel T. L. Bright A. D. (2004). “Applications of the concepts of values and attitudes in human dimensions of natural resources research,” in *Society and Natural Resources: A Summary of Knowledge*, eds Manfredo M. J. Vaske J. J. Bruyere B. L. Field D. R. Brown P. (Jefferson City, MO: Modern Litho), 271–282.

[B31] Manning R. E. (2007). *Studies in Outdoor Recreation: Search and Research for Satisfaction*, 3rd Edn Corvallis, OR: Oregon State University Press.

[B32] Marin L. D. Newman P. Manning R. Vaske J. J. Stack D. (2011). Motivation and acceptability norms of human-caused sound in Muir Woods National Monument. *Leis. Sci.* 33 147–161. 10.1080/01490400.2011.550224

[B33] McDonald C. D. Baumgartner R. M. Iachan R. (1995). *National Park Service Aircraft Management Studies.* USDI Report No. 94-2 Denver, CO: National Park Service.

[B34] Monz C. A. (2009). Climbers’ attitudes toward recreation resource impacts in the Adirondack Park’s Giant Mountain Wilderness. *Int. J. Wilderness* 15 26–33.

[B35] National Park Service [NPS] (2000). *Director’s Order #47: Soundscape Preservation and Noise Management.* Available at: http://www.nps.gov/policy/DOrders/DOrder47.html [accessed November 24, 2011].

[B36] National Park Service [NPS] (2006). *National Park Service Management Policies.* Washington, DC: U.S. Government Printing Office.

[B37] Newman P. Manning R. Trevino K. (2010). From landscapes to soundscapes: introduction to the special issue. *Park Sci.* 26 2–5.

[B38] Payne S. R. (2008). Are perceived soundscapes within urban parks restorative? *J. Acoust. Soc. Am.* 123 5519–5524. 10.1121/1.2935525

[B39] Pedersen E. Berg F. Bakker R. Bouma J. (2009). Response to noise from modern wind farms in the Netherlands. *J. Acoust. Soc. Am.* 126 634–643. 10.1121/1.3160293 19640029

[B40] Perloff R. M. (2003). *The Dynamics of Persuasion: Communication and Attitudes in the 21st Century*, 2nd Edn Mahwah, NJ: Lawrence Erlbaum Associates.

[B41] Petty R. E. Cacioppo J. T. (1986). *Communication and Persuasion: Central and Peripheral Routes to Attitude Change.* New York, NY: Springer-Verlag 10.1007/978-1-4612-4964-1

[B42] Petty R. E. Wegener D. (2008). “Matching versus mismatching attitude functions: implications for scrutiny of persuasive messages,” in *Attitudes Their Structure, Function and Consequences: Key Readings in Social Psychology*, eds Fazio R. H. Petty R. E. (New York, NY: Psychology Press).

[B43] Petty R. E. Wegener D. T. (1998). Matching versus mismatching attitude functions: implications for scrutiny of persuasive messages. *Pers. Soc. Psychol. Bull.* 24 227–240. 10.1177/0146167298243001

[B44] Pheasant R. J. Horoshenkov K. Watts G. Barret B. T. (2008). The acoustic and visual factors influencing the construction of tranquil space in urban and rural environments tranquil spaces-quiet places? *J. Acoust. Soc. Am.* 123 1446–1457. 10.1121/1.2831735 18345834

[B45] Pheasant R. J. Watts G. R. (2015). Towards predicting wildness in the United Kingdom. *Landsc. Urban Plan.* 133 87–97. 10.1016/j.landurbplan.2014.09.009

[B46] Pilcher E. J. Newman P. Manning R. E. (2009). Understanding and managing experimental aspects of soundscapes at Muir Woods National Monument. *Environ. Manage.* 43 425–435. 10.1007/s00267-008-9224-1 19020928

[B47] Reed S. E. Boggs J. L. Mann J. P. (2012). A GIS tool for modeling anthropogenic noise propagation in natural ecosystems. *Environ. Modell. Softw.* 37 1–5. 10.1016/j.envsoft.2012.04.012

[B48] Shelby B. Heberlein T. A. (1986). *Carrying Capacity in Recreational Settings.* Corvallis, OR: Oregon State University Press.

[B49] Stack D. W. Peter N. Manning R. E. Fristrup K. M. (2011). Reducing visitor noise levels at Muir Woods National Monument using experimental management. *J. Acoust. Soc. Am.* 129 1375–1380. 10.1121/1.3531803 21428501

[B50] Taff B. D. Newman P. Pettebone D. White D. D. Lawson S. R. Monz C. (2013). Dimensions of alternative transportation experience in Yosemite and Rocky Mountain National Parks. *J. Transp. Geogr.* 30 37–46. 10.1016/j.jtrangeo.2013.02.010

[B51] Taff D. Newman P. Lawson S. R. Bright A. Marin L. Gibson A. (2014). The role of messaging on acceptability of military aircraft sounds in Sequoia National Park. *Appl. Acoust.* 84 122–128. 10.1016/j.apacoust.2013.09.012

[B52] Tarrant M. A. Haas G. E. Manfredo M. J. (1995). Factors affecting visitor evaluations of aircraft overflights of wilderness areas. *Soc. Nat. Resour.* 8 351–360. 10.1080/08941929509380927

[B53] Vaske J. J. (2008). *Survey Research and Analysis: Applications in Parks, Recreation and Human Dimensions.* State College, PA: Venture Publishing.

[B54] Watson D. Clark L. A. Tellegen A. (1988). Development and validation of brief measures of positive and negative affect: the PANAS scales. *J. Pers. Soc. Psychol.* 54 1063–1070. 10.1037/0022-3514.54.6.1063 3397865

[B55] Weinzimmer D. Newman P. Taff D. Benfield J. Lynch E. Bell P. (2014). Human responses to simulated motorized noise in national parks. *Leis. Sci.* 36 251–267. 10.1080/01490400.2014.888022

[B56] White D. D. Aquino J. F. Budruk M. Golub A. (2011). Visitors’ experiences of traditional and alternative transportation in Yosemite National Park. *J. Park Recreation Adm.* 29 38–57.

[B57] Wood W. (2000). Attitude change: persuasion and social influence. *Annu. Rev. Psychol.* 51 539–570. 10.1146/annurev.psych.51.1.539 10751980

[B58] Ziegler R. Dobre B. Diehl M. (2007). Does matching versus mismatching message content to attitude functions lead to biased processing? The role of message ambiguity. *Basic Appl. Soc. Psychol.* 29 269–278. 10.1080/01973530701503366

